# Von Willebrand factor:antigen and ADAMTS-13 level, but not soluble P-selectin, are risk factors for the first asymptomatic deep vein thrombosis in cancer patients undergoing chemotherapy

**DOI:** 10.1186/s12959-020-00247-6

**Published:** 2020-11-11

**Authors:** Budi Setiawan, Cecilia Oktaria Permatadewi, Baringin de Samakto, Ashar Bugis, Ridho M. Naibaho, Eko Adhi Pangarsa, Damai Santosa, Catharina Suharti

**Affiliations:** 1 Division of Hematology and Medical Oncology, Department of Internal Medicine, Medical Faculty of Diponegoro University and Dr. Kariadi Hospital, Semarang, Indonesia; 2 Department of Internal Medicine, Medical Faculty of Diponegoro University and Dr. Kariadi Hospital, Semarang, Indonesia; 3 Fellow in Hematology and Medical Oncology, Department of Internal Medicine, Medical Faculty of Mulawarman University, Parikesit General Hospital, Kutai Kartanegara, Indonesia

**Keywords:** Deep vein thrombosis, sP-selectin, vWF, ADAMTS-13, Cancer, Chemotherapy

## Abstract

**Background:**

There is a high incidence of deep vein thrombosis (DVT) among cancer patients undergoing chemotherapy. Chemotherapy-induced vascular endothelial cell activation (VECA) is characterized by increased plasma levels of von Willebrand factor (vWF) and soluble P-selectin (sP-selectin), leading to the activation of endothelial cells and signaling cascades. The biological role of a disintegrin-like and metalloproteinase with thrombospondin type 1 motif, member 13 (ADAMTS-13) is to control the activity of vWF and consequently the risk of thrombosis. The objective of this study was to investigate the roles of sP-selectin, vWF, and ADAMTS-13 as risk factors for the first episode of DVT in cancer patients undergoing chemotherapy.

**Methods:**

This prospective cohort study was conducted at Dr. Kariadi Hospital, Indonesia, on 40 cancer patients. Prechemotherapy (baseline) and postchemotherapy sP-selectin, vWF antigen (vWF:Ag), and ADAMTS-13 plasma levels were determined with ELISAs before and 3 months after chemotherapy. The clinical characteristics of the patients, cancer type, cancer stage, chemotherapy regimen, ABO blood type, D-dimer level and Khorana risk score were also analyzed using logistic regression. Patients were observed for the possibility of developing DVT during chemotherapy.

**Results:**

DVT was confirmed in 5 patients (12.5%) after a period of 3 months. In patients with DVT, sP-selectin and vWF were significantly higher while ADAMTS-13 was lower than in their counterparts. The levels of baseline vWF:Ag and ADAMTS-13, with cut-off points ≥ 2.35 IU/mL and ≤ 1.03 IU/mL, respectively, were found to independently predict the incidence of DVT. In the multivariate logistic regression analysis, the relative risk (RR) for DVT in patients with high vWF:Ag was 3.80 (95% CI 1.15–12.48, *p* = 0.028), and that for patients with low ADAMTS-13 was 2.67 (95% CI 1.22–23.82, *p* = 0.005). The vWF:Ag/ADAMTS-13 ratio and both vWF:Ag and ADAMTS-13 dynamics during treatment were also able to differentiate those with prospective DVT. However, sP-selectin and other covariates showed no statistical significance.

**Conclusion:**

We found that prechemotherapy plasma levels of vWF:Ag ≥ 2.35 IU/mL and ADAMTS-13 ≤ 1.03 IU/mL are independent risk factors for DVT incidence among cancer patients.

## Introduction

Deep vein thrombosis (DVT) and pulmonary embolism (PE), collectively referred to as venous thromboembolism (VTE), are major burdens in cancer that could lead to significant morbidity, prolonged hospitalization, increased treatment costs and a notable cause of death for patients [1]. Of all medical conditions, cancer is the strongest risk factor for thrombosis (up to 50-fold relative risk (RR) in the first 6 months after diagnosis) [2]. DVT embolization from the lower extremities is considered the main cause of PE. Asymptomatic patients and those with untreated DVT are reported to have a 50% risk of developing PE within 3 months after disease onset, with a substantial mortality risk [3]. Thrombosis is indeed the second leading cause of death in cancer patients receiving chemotherapy, following death caused by cancer progression itself [4]. The number of cancer patients has continuously increased worldwide, and the adequate treatment of cancer-associated thromboembolism is therefore particularly important [1, 5].

The propensity of thrombosis in cancer patients is multifactorial and includes various types of patient-, tumor-, and treatment-related risk factors and biomarkers [1, 6]. This study aimed to identify the acquired risk factors for DVT in cancer patients, particularly those who received chemotherapy. Heit et al. [7] performed a population-based study and found that cancer patients had a higher risk of developing VTE than those without cancer (RR 4.1), and the RR increased to 6.5 with chemotherapy. In general, the cytotoxic effects of chemotherapy may lead to endothelial damage. Biochemically, chemotherapy-induced vascular endothelial cell activation (VECA) is indicated by an increasing number of endothelial cells in the circulation and other plasma markers, von Willebrand factor (vWF) in the plasma, adhesion molecules, and selectins. It is also highly probable that the liberation of such biochemical markers into the circulation by injured endothelial cells may precede alterations in their functions [8].

Selectins are adhesion molecules that mediate calcium-dependent cell-to-cell interactions among leukocytes (L-selectin), platelets (P-selectin) and endothelial cells (P- and E-selectins) [9]. P-selectin is constitutively expressed in endothelial cells of the lung and the choroid plexus, megakaryocytes, and platelets and is stored within the Weibel-Palade bodies (WPBs) of endothelial cells or the alpha granules of platelets [10]. The binding of P-selectin to its specific counterreceptor, P-selectin specific ligand-1 (PSGL-1), on the surface of leukocytes and platelets increases tissue factor expression and initiates various procoagulant activities [11]. P-selectin can be identified as a circulating plasma protein, sP-selectin, which is shed from activated platelets and endothelial cells, and its function remains enigmatic [10, 12]. Some researchers have found that sP-selectin is a better biomarker of VTE than D-dimer [13].

The platelet-adhesive blood coagulation protein vWF is synthesized mainly in vascular endothelial cells and megakaryocytes and stored in WPBs and the alpha granules of platelets in the form of “ultra large” vWF (UL-vWF). vWF is a multimeric protein that promotes platelet adhesion and aggregation under high shear stress conditions. This glycoprotein also acts as a carrier for coagulation factor VIII and plays a bifunctional role in primary and secondary hemostasis [14]. Several studies have also demonstrated increased plasma vWF levels in cancer patients [15]. A disintegrin and metalloproteinase with a thrombospondin type 1 motif, member 13 (ADAMTS-13) specifically cleaves vWF multimers and regulates their prothrombotic properties, producing a smaller and less active vWF subunit [16]. ADAMTS-13 is secreted by hepatic stellate cells [17] and endothelial cells [18]. Data on ADAMTS-13 are highly ambiguous. Lancelotti et al. [19] revealed that decreased ADAMTS-13 activity was related to VTE, while Mazetto et al. [20] reported the opposite. The interrelationship between sP-selectin to vWF antigen (vWF:Ag) and vWF:Ag to ADAMTS-13 and the calculated ratios are also of particular interest.

Based on the abovementioned reasons, this single-center prospective study was designed to investigate the roles of sP-selectin, vWF, and ADAMTS-13 as risk factors for DVT incidence in cancer patients undergoing chemotherapy. As previously hypothesized, increased levels of soluble P-selectin indicate platelet activation [12, 13, 21], while vWF and ADAMTS-13 could be regarded as markers of endothelial disruption [14, 18, 21]. This study aimed to provide a better understanding of the pathophysiology of chemotherapy-induced thrombosis and contribute to an early primary evaluation for cancer patients at risk of DVT.

## Methods

### Study setting

This prospective cohort study was performed at Dr. Kariadi Hospital, the main teaching hospital for the Medical Faculty of Diponegoro University, Semarang, Indonesia. This hospital is a tertiary referral hospital for all patients with cancer in Central Java province. The endpoint of this study was objectively confirmed DVT within 3 months after first-line chemotherapy was given, without verification of either venous or arterial thrombosis at the time of enrollment. All patients were evaluated by the Wells’ score probability test and D-dimer level. Patients with a positive D-dimer level (≥500 ng/mL) and/or a high probability Wells’ score (≥2) were referred to undergo a vascular duplex ultrasound. Short-cycle chemotherapy was administered as outpatient therapy, while a protocol consisting of more than 2 days of chemotherapy was administered as inpatient therapy. None of the patients received primary prophylaxis with anticoagulation because the patients who were included in this study had an Eastern Cooperative Oncology Group (ECOG) performance status ≥2 to maintain their active mobilization. This study was approved by the Internal Review Board of Dr. Kariadi Hospital. Written informed consent was obtained from all subjects. This study was conducted in accordance with the Declaration of Helsinki.

### Patient selection and data collection

**Fig. 1:**
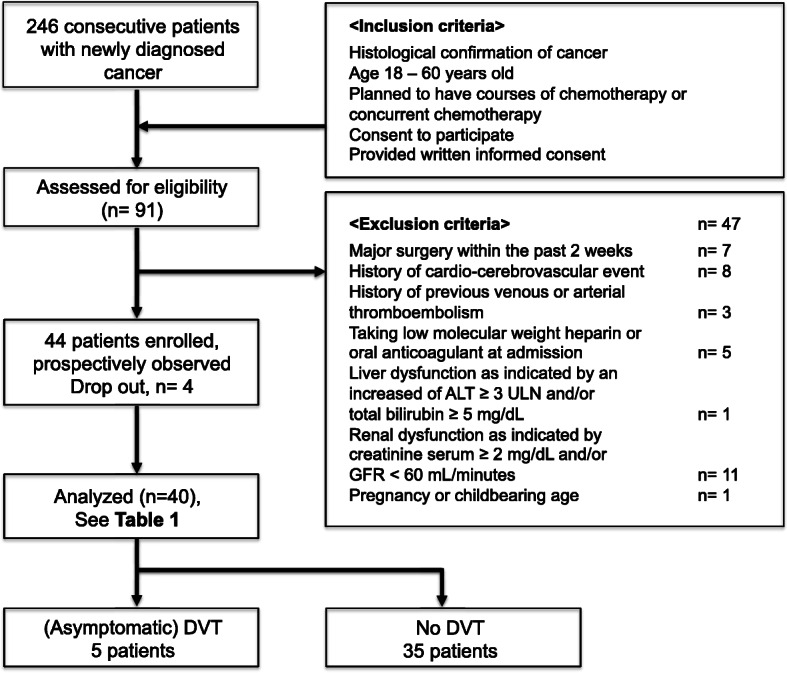
Patient selection and enrollment of the cohort study. A total of 246 patients diagnosed with cancer between November 2016 and February 2017 were initially evaluated for study enrollment. Of those, only 44 fulfilled the inclusion and exclusion criteria. Four patients were excluded due to various reasons, leaving only 40 cancer patients undergoing chemotherapy. Five patients (12.5%) developed asymptomatic DVT at the end of the observation period. Abbreviations: ALT, alanine transaminase; DVT, deep vein thrombosis; GFR, glomerular filtration rate; ULN, upper limit of normal value

From November 2016 to February 2017, a total of 246 consecutive and unselected newly diagnosed cancer patients were screened. Forty consecutive patients with active cancers undergoing chemotherapy were enrolled (see Fig. 1 for patient enrollment). The inclusion criteria were as follows: newly diagnosed cancer with a histological confirmation, age over 18 years, ECOG performance status ≤2, consent to participate, and signed written informed consent. The exclusion criteria were as follows: overt bacterial or viral infection within the last 2 weeks, hepatic and renal dysfunction, venous or arterial thromboembolism within the last 3 months and the use of an anticoagulant, aspirin or statin and surgery or radiotherapy within the last 2 weeks. Inherited VTE risk factors were not taken into consideration. The probability test for Wells’ score (which will be described later) should indicated “DVT unlikely” for all participants.

Before this study was conducted, all patients had been informed about the study’s details in an individual interview. Then, anamneses on patients’ cancer history, tumor site, tumor histology and tumor stage were documented. Patients who met the inclusion criteria were selected as study subjects. Samples were examined twice (before and after chemotherapy) to measure the plasma levels of sP-selectin, vWF:Ag and ADAMTS-13. Age, sex, smoking history, ABO blood group, body mass index, DVT-related history, diabetes, hypertension, history of atherosclerosis, drug, type of cancer, and chemotherapy regimen were carefully recorded.

### Treatment and follow-up

All patients received chemotherapy or chemoradiotherapy. Cisplatin/carboplatin-based chemotherapy included gemcitabine, paclitaxel, docetaxel or pemetrexed. Fluorouracil-based chemotherapy was used in colorectal cancer patients as a component of the FOLFOX/FORFIRI/de Gramont protocol with or without bevacizumab or cetuximab. Anthracycline-based chemotherapy was used in several patients, including those with acute myeloid leukemia (AML) who received a 3 + 7 protocol containing daunorubicin or doxorubicin in the R-CHOP protocol. The patients were monitored for 3 months and evaluated during their follow-up visits at the Hematology and Medical Oncology Clinic by the attending staff. In the first, second and third months, evaluations of DVT occurrence were conducted clinically by examinations as well as the Wells’ pretest probability model to assess DVT. If the Wells’ score was ≥2, color duplex sonography was performed to establish the occurrence of DVT. Otherwise, color duplex sonography was conducted at the end of the third month.

### Outcome measure: the deep vein thrombosis

The endpoint of this study was the occurrence of DVT, either asymptomatic, symptomatic or fatal VTE, confirmed by a duplex ultrasound. Color duplex sonography was performed at the Radiology Department of Dr. Kariadi Hospital, Semarang, Indonesia. Patients with clinically suspected DVT and a Wells’ score ≥ 2 were assessed for DVT by using a Logiq 7 Pro US imaging system (Logiq 7 Pro; GE Healthcare, USA) with a 7–10 Hz linear probe. The diagnosis of DVT was based either on the presence of a noncompressible segment (compression ultrasound test – CUS) or flow impairment on color Doppler imaging. Patients were examined for both proximal (popliteal, femoral, and common femoral veins) and distal (peroneal and tibial veins) DVT. The first duplex ultrasound was performed within the first 7 days after inclusion and then during chemotherapy or if the Wells’ score was ≥2 or at the final observation in the third month.

### Prediction score

Two prediction scores were used in this study: the Khorana risk score and Wells’ score. For each patient, we calculated the Khorana risk score to stratify the risk of VTE in cancer patients undergoing chemotherapy [22]. Patients were assigned to three risk categories for VTE: low risk = 0, intermediate risk = 1–2, and high risk ≥3. A Wells’ score of 1 point each was given for active cancer, paralysis, paresis, recent plaster immobilization of the lower limb, recently bedridden for > 3 days, major surgery in the past 4 weeks, localized tenderness along the distribution of the deep venous system, entire leg swollen, calf swelling > 3 cm compared to the asymptomatic leg, pitting edema and collateral superficial veins. Two points were subtracted from this score for an alternative diagnosis as likely DVT or more likely than DVT. A score of 3 or higher suggests that DVT is likely, the patient should receive a diagnostic US, and the results should be documented [23].

### Laboratory measurements

Venous blood specimens were collected by sterile and atraumatic antecubital venipuncture into citrate vacutainer tubes (SST 5 mL) containing 0.5 mL of liquid anticoagulant. Measurements of plasma sP-selectin levels were carried out using a recombinant human P-selectin/CD62P immunoassay (catalog number ADP3; R&D Systems, Inc., 614 McKinley Place NE, Minneapolis, MN 55413, USA) [24]. vWF antigen was measured using an ELISA (catalog number 885_BcSD20121001; Sekisui Diagnostic, LLC, 500 West Avenue, Stamford) [25]. Plasma ADAMTS-13 levels were measured with a Quantikine ELISA human ADAMTS-13 immunoassay (R&D Systems, Inc., 614 McKinley Place NE, Minneapolis, MN 55413, USA) [26].

Blood samples were collected at the following time points: (i) baseline, before initial chemotherapy, and (ii) 3 months after initial chemotherapy. Samples for ELISA were immediately centrifuged at 2500 g for 15 min, and plasma samples were aliquoted, coded and stored at − 80 °C until the assays were performed. Samples were prepared according to the manufacturer’s instructions, and plates were read on an ELx808 plate reader (Biotek, Vermont) at a wavelength of 450 nm. The results were converted to total protein using a bicinchoninic acid (BCA) assay (Pierce Rockford, Illinois) and are reported as ng/mg total protein. Measurements were performed in a blinded manner. All samples were assayed in duplicate, and those showing values above the standard curve were retested with appropriate dilutions.

### Cut-off points and normal reference values

sP-selectin, vWF:Ag and ADAMTS-13 activity cut-off points were determined based on fold changes. The minimum detectable dose for sP-selectin was 0.5 ng/mL, and the range in citrate plasma was 20–44 ng/mL [24]. With regard to vWF:Ag, the reported mean level was 1.03 ± 0.3 IU/mL in men and 1.08 ± 0.4 IU/mL in women [27]. According to Green et al., the mean vWF:Ag/ADAMTS-13 ratio is 1.05 ± 0.30 IU/mL [28]. The ADAMTS antigen level in noncancer patients was 0.70–1.42 IU/mL (median 1.08 IU/mL) [29]. According to the manufacturer, serum ADAMTS-13 levels range from 0.51–1.64 IU/mL (Quantikine ELISA Human ADAMTS Immunoassay, R&D Systems, Inc.) [26]. The cut-off values for sP-selectin, vWF:Ag, and ADAMTS-13 were set at 105.5 ng/mL, 2.35 IU/mL, and 1.03 IU/mL, respectively, according to the 75th percentiles of the levels observed in this cohort.

### Response to chemotherapy and follow-up

All patients who underwent chemotherapy or chemoradiotherapy were followed up for 3 months. After obtaining informed consent, patients were evaluated either during routine visits at the Hematology and Medical Oncology Outpatient Clinic or at the medical ward every pre- and postchemotherapy cycle. The performance status, chemotherapy eligibility and Wells’ score were assessed at each visit. DVT occurring after enrollment was documented as a new event, first lifetime thrombosis. Median differences between the baseline and postchemotherapy levels of each independent variable were calculated and are reported as positive or negative delta values.

### Statistical analysis

Quantitative variables were examined for normality with a Shapiro-Wilk test. Continuous variables are summarized as medians (minimum-maximum), whereas categorical data are described as absolute frequencies and percentages. Clinical and laboratory parameters, including sP-selectin, vWF:Ag, and ADAMTS-13 levels and their delta values between DVT and non-DVT subjects were compared using a nonparametric Mann-Whitney test. Correlations between two continuous variables were evaluated with Spearman’s rank correlation coefficient. To create positive and negative predictive values, we computed a logistic regression model with three independent variables together with other possible confounding factors. Dichotomous variables were created for all patients in our data set by comparing the probability of DVT associated with the selected cut-off point.

Stepwise multiple regression analysis was used to examine differences in sP-selectin, vWF:Ag, and ADAMTS-13 levels between baseline and postchemotherapy. The vWF:ADAMTS-13 ratio and other potential determinants, such as age, sex, smoking history, cardiovascular risk factors (such as overweight/obesity, hypertension and diabetes), Khorana risk score, D-dimer, chemotherapy regimen, and other biological factors (type of cancer and stage of disease) were also included. First, all potential predictors were entered simultaneously into a multivariate logistic regression model that was reduced using a backward selection method as the final step. Multivariate logistic regression was adjusted for all independent predictors, and only variables with correlations to the outcome (defined as *p* < 0.25 in the univariate model) were included. We generated the final multivariate model for DVT outcome using a backward stepwise approach, and *p* < 0.05 from the likelihood ratio test was used to exclude excess factors. The precision of the specified model to detect DVT incidents was quantified by the Hosmer-Lemeshow goodness-of-fit statistic, where a value greater than 0.05 indicates adequate calibration for the corresponding area under the receiver operating characteristic (ROC) curve [30]. The Statistical Package for Social Sciences (IBM v. 21; SPSS, Inc., USA) was used for all data analyses. All tests with *p* values < 0.05 were considered statistically significant.

## Results

### Patient characteristics and anatomical distribution of DVT

Table 1 shows the characteristics of the study population. Overall, 55% of patients were male and 45% were female. One patient died prior to chemotherapy during week 6. The median age of the patients was 49 (20–71) years, and the mean body mass index was 19.4 (15.2–22.6) kg/m^2^. The main cancer entities were colorectal (45%) and cervical (15%) cancers. Distant metastasis was found in 42.5% of patients. During the observation period, all patients received chemotherapy. Seven patients received chemotherapy as a radiosensitizer, and the remainder received chemotherapy as either neoadjuvant, adjuvant or palliative chemotherapy. At enrollment, 27.5% of patients were treated as outpatients and 63.5% as inpatients; however, patient mobilization was maintained during the study period. None of the study participants used an erythropoietic-stimulating agent at study inclusion.

**Table 1:** Clinical characteristics of study participants

Characteristics	All patients (***n*** = 40)	No DVT after enrollment (***n*** = 35)	DVT after enrollment (***n*** = 5)
Age at study entry (years), median (minimum – maximum)	49 (20–71)	49 (21–71)	42 (20–59)
Sex, n (%)			
Male	22 (55%)	20 (57.2%)	2 (40%)
Female	18 (45%)	15 (42.8%)	3 (60%)
Blood group, n (%)			
O blood group	8 (20%)	8 (22.8%)	0 (0%)
Non-O blood group (A, B, AB)	32 (80%)	27 (77.1%)	5 (100%)
Body mass index (kg/m^2^)	19.4 (15.2–22.6)	19.4 (16.5–22.6)	19.7 (15.2–20.2)
Underweight	11 (27.5%)	9 (25.7%)	2 (40%)
Normoweight	29 (72.5%)	26 (74.3%)	3 (60%)
Overweight/obese	0 (0%)	0 (0%)	0 (0%)
Primary site of cancer, n (%)			
Colorectal	18 (45%)	17 (48.6%)	1 (20%)
Genitourinary	6 (15%)	3 (8.6%)	3 (60%)
Pancreas	3 (7.5%)	2 (5.7%)	1 (20%)
Lung	2 (5%)	2 (5.7%)	0 (0%)
Upper gastrointestinal tract	2 (5%)	2 (5.7%)	0 (0%)
Leukemia and lymphoma	2 (5%)	2 (5.7%)	0 (0%)
Others	7 (17.5%)	7 (20%)	0 (0%)
Stage at diagnosis, n (%)			
Localized	23 (57.5)	21 (60%)	2 (40%)
Advanced/metastasis	17 (42.5)	14 (40%)	3 (60%)
Chemotherapy regimen			
de Gramont/FOLFOX/FOLFIRI	10 (25%)	9 (25.7%)	1 (20%)
Paclitaxel + Cisplatin/Carboplatin	7 (17.5%)	6 (17.1%)	1 (20%)
FOLFOX + Bevacizumab	4 (10%)	4 (11.4%)	0 (0%)
FOLFIRI + Cetuximab	3 (7.5%)	3 (8.6%)	0 (0%)
de Gramont + Bevacizumab	1 (2.5%)	1 (2.8%)	0 (0%)
Doxorubicin-Ifosfamide	2 (5%)	2 (5.7%)	0 (0%)
Cisplatin-Fluorouracil	2 (5%)	2 (5.7%)	0 (0%)
Cisplatin+XRT	3 (7.5%)	3 (8.6%)	2 (40%)
Gemcitabine + Cisplatin/Carboplatin	3 (7.5%)	3 (8.6%)	0 (0%)
UK-ALL protocol	1 (2.5%)	1 (2.8%)	0 (0%)
Gemcitabine	1 (2.5%)	0 (0%)	1 (20%)
Paclitaxel	1 (2.5%)	1 (2.8%)	0 (0%)
R-CHOP	1 (2.5%)	1 (2.8%)	0 (0%)
3 + 7 protocol	1 (2.5%)	1 (2.8%)	0 (0%)
Radiotherapy	7 (17.5%)	5 (14.3%)	2 (40%)
Laboratory parameters			
Hemoglobin (g/dL)	11.1 (5.9–16.3)	10.9 (5.9–16.3)	11.2 (10.0–12.5)
Leukocytes (× 10^3^/μL)	9.2 (3.9–99.1)	8.8 (3.88–99.1)	11.9 (6.3–50.3)
Platelets (×10^3^/μL)	340.0 (51.0–766.0)	330.2 (51.0–766.0)	449.5 (200–561)
D-dimer (ng/mL)	1.859.8 (230–11,450)	1370.12 (230–11,450)	3920 (460–7140)
Khorana risk score			
Low risk	0 (0%)	0 (0%)	0 (0%)
Intermediate risk	23 (57.5)	20 (57.2%)	3 (60%)
Very high risk	17 (42.5)	15 (42.8%)	2 (40%)

Patients were followed up regularly for a minimum of 4 occasions, and during this time, no patient developed clinical signs and symptoms of DVT, and Wells’ score was < 2 in all patients. At the end of the observation period, duplex ultrasound was performed in all participants, and objective findings compatible with DVT were found in 5 patients (12.5%). Two patients had proximal thrombosis involving the femoral vein, whereas the other 3 patients were diagnosed with leg vein thrombosis. Thrombosis was seen more often in males than in females (3 vs. 2 patients). DVT was “asymptomatic” in all patients and subsequently treated with an anticoagulant according to local practice.

### Plasma concentration of D-dimer, the Khorana risk score, cancer stage and ABO blood group in cancer patients with and without DVT

D-dimer levels at admission were higher in cancer patients who developed DVT than in those who did not (*p* = 0.013). D-dimer showed a positive correlation with sP-selectin (*r* = 0.536, *p* < 0.001) and vWF:Ag (*r* = 0.398, *p* = 0.011) but no significant correlation with ADAMTS-13 (*r* = − 0.226, *p* = 0.162). According to the Khorana risk score, the majority (57.5%) of the study population had an intermediate score, while 42.5% had a high score, and 0% had a low score. The Khorana risk score was assessed in all patients and showed no difference in risk group distribution between patients with and without DVT.

With increasing cancer stage, both sP-selectin and vWF:Ag levels were increased, whereas ADAMTS-13 levels were decreased at the time of inclusion. No statistically significant difference was observed between sP-selectin, vWF:Ag, and ADAMTS-13 levels in study participants with cancer sorted by stage or the Khorana risk score (*p* > 0.05 for all, Mann-Whitney U test). However, this method did not appear to discriminate patients when taking into account the proportion of patients with high risk scores. Detailed information is given in Table 2.

**Table 2:** sP-selectin, vWF:Ag and ADAMTS-13 levels according to cancer stage and the Khorana risk score category

Covariate	sP-selectin (ng/mL)	vWF:Ag (IU/mL)	ADAMTS-13 (IU/mL)
Local disease (*n* = 23)	69.3 (39.10–230.60)	1.36 (0.53–3.01)	0.97 (0.76–1.22)
Advanced/metastasis (*n* = 17)	88.3 (31.30–185.40)	1.68 (0.37–3.75)	0.85 (0.42–1.33)
*p*^§^	0.479	0.066	0.671
Low/intermediate Khorana risk score (*n* = 22)	68.8 (37.30–145.10)	1.15 (0.37–2.97)	0.92 (0.42–1.33)
High/very high Khorana risk score (*n* = 18)	99.0 (31.30–230.60)	1.76 (0.80–3.75)	0.85 (0.45–1.21)
*p* ^§^	0.203	0.626	0.149

*NOTE*: ^§^Mann-Whitney U Test

The ABO blood group has a significant influence on vWF:Ag levels, where the O blood group has lower levels than the non-O groups (median 0.73 U/mL vs. 1.4 U/mL, *p* < 0.001), as shown in Table 3. In contrast, the level of ADAMTS-13 was higher in the O blood group than in the non-O groups (median 0.99 U/mL vs. 0.84 U/mL, *p* = 0.062). With regard to the sP-selectin level, there was no significant difference between the ABO blood groups (*p* = 0.310).

**Table 3:** Comparison of vWF:Ag and ADAMTS levels in patients according to ABO blood groups

Covariate	Group O (***n*** = 8)	Group non-O (***n*** = 32)	***p***^**§**^
vWF: Ag (IU/mL)	0.73 (0.37–0.95)	1.4 (0.65–3.75)	< 0.001
ADAMTS-13 (IU/mL)	0.99 (0.76–1.33)	0.84 (0.42–1.22)	0.062
sP-selectin (ng/mL)	68.3 (39.1–104.2)	85.4 (31.3–230.6)	0.310

Data are presented as the median (minimum-maximum range)

*NOTE*: ^§^Mann-Whitney U Test

### Plasma concentrations of vWF:Ag and ADAMTS-13 in cancer patients with and without DVT

**Fig. 2:**
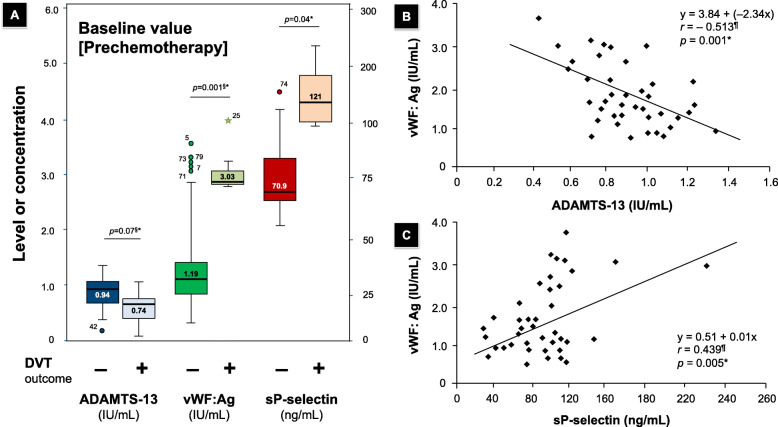
**a**. Prechemotherapy (baseline) plasma levels of ADAMTS-13, vWF:Ag and sP-selectin in 40 cancer patients stratified based on the occurrence of DVT at the end of the observation period. Boxplot data are presented as the median value (minimum-maximum range), and the *p* value represents the difference between groups. **b**. Correlation coefficient, scatterplot and regression line between the vWF:Ag level and ADAMTS activity. **c**. Correlation coefficient, scatterplot and regression line between the vWF:Ag level and soluble P-selectin. NOTE: ^§^Mann-Whitney U test; ^¶^Spearman’s rank test; *Statistically significant at *p* < 0.05

In this study, the baseline levels of vWF:Ag measured by ELISA were relatively high compared to those in normal healthy individuals [27] and markedly increased after chemotherapy. The median baseline and postchemotherapy vWF:Ag levels were 1.30 (0.37–3.75) and 1.50 (0.72–3.97) IU/mL, respectively (Table 4). The levels of vWF:Ag were higher in patients with prospective DVT than in those without DVT (3.03 vs. 1.19 IU/mL, *p* = 0.001). The median level of ADAMTS-13 was similar to the normal reference at both baseline and postchemotherapy. However, as shown in Fig. 2, the baseline level of ADAMTS-13 was lower in patients with DVT than in those without DVT.

**Table 4:** sP-selectin, vWF:Ag, and ADAMTS levels, vWF/ADAMTS-13 ratios and respective delta values between baseline and postchemotherapy (*n* = 40)

Covariate	Baseline	Postchemotherapy	Difference in the median (delta value)	***p*** ^**§**^
sP-selectin (ng/mL)	81.95 (31.30–230.60)	92.5 (40.9–278.3)	+ 7.70 (1.0–88.1)	< 0.001
vWF:Ag (IU/mL)	1.30 (0.37–3.75)	1.50 (0.72–3.97)	+ 0.21 (0.02–1.22)	< 0.001
ADAMTS-13 antigen (IU/mL)	0.86 (0.42–1.33)	0.94 (0.31–1.64)	+ 0.03 (−0.39–0.36)	0.026
vWF:Ag/ ADAMTS-13 ratio	1.46 (0.39–8.33)	1.7 (0.56–12.80)	+ 0.17 (−1.24–4.47)	0.001

Data are presented as the median (minimum-maximum range)

*NOTE*: ^§^Wilcoxon signed-rank test (between baseline and postchemotherapy)

We also observed that the median level of ADAMTS-13 slightly increased over time after chemotherapy (0.86 to 0.94, delta value + 0.03; *p* = 0.026; see Table 2). The dynamics of ADAMTS-13 can differentiate cancer patients who will develop DVT with a further reduction in ADAMTS-13 during chemotherapy, creating a negative delta value (Fig. 2a). High vWF:Ag values were not necessarily associated with the occurrence of DVT, and interestingly, we found that those who developed DVT had a higher vWF/ADAMTS-13 ratio than their counterparts. DVT was observed in patients with high vWF levels and low ADAMTS-13 levels. Correlation analyses between ADAMTS-13 and vWF activities were conducted and revealed a significant inverse correlation (*r* = − 0.513, *p* = 0.001), as shown in Fig. 2b.

### Plasma vWF:Ag/ADAMTS-13 ratios in cancer patients with and without DVT

**Fig. 3:**
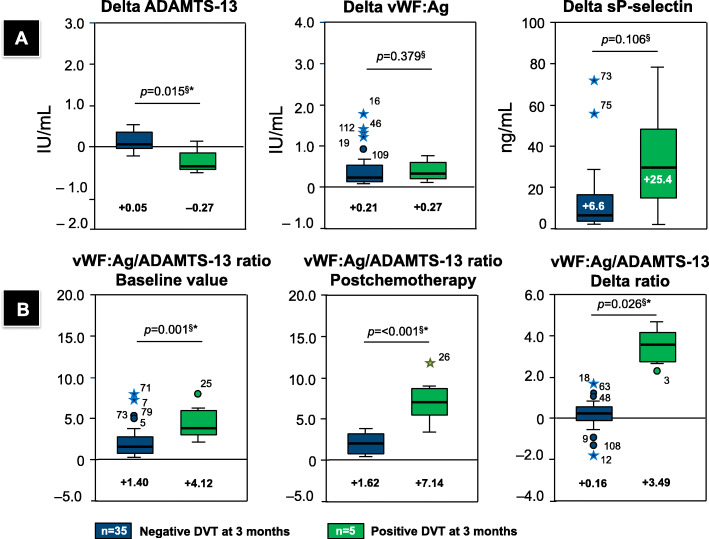
**a**. Differences in each predictor (1. ADAMTS-13, 2. vWF:Ag, and 3. sP-selectin). Shifts in prechemotherapy (baseline) vs. postchemotherapy levels are reported as the delta values for prospective DVT. **b**. Respective vWF:ADAMTS-13 ratios (1. Prechemotherapy, 2. Postchemotherapy, and 3. Delta value for the ratio). NOTE: ^§^ Mann-Whitney U test; *Statistically significant at *p* < 0.05

Differences in the overall direction and dynamics for both vWF:Ag and ADAMTS-13 during the course of chemotherapy at baseline and postchemotherapy (delta value) can also illustrate the “risk” of developing DVT by dividing patients according to the ratio of vWF:Ag/ADAMTS-13 at the end of the observation period. A negative correlation between vWF:Ag and ADAMTS-13 was observed (0.513, *p* = 0.001), corresponding to the wide gap ratio in certain patients during the course of chemotherapy. A high vWF:Ag/ADAMTS-13 ratio and either increased vWF:Ag or decreased ADAMTS-13 were closely related to DVT occurrence. Figure 3b shows that overall, the highest deviation of the reverse correlation between vWF:Ag and ADAMTS-13 (+ 3.49 vs. + 0.16, *p* = 0.025) will differentiate cancer patients who will develop DVT within the first 3 months following chemotherapy.

The vWF:Ag/ADAMTS-13 ratio was significantly higher in cancer patients with DVT than in those without (+ 4.12 vs. + 1.40, *p* = 0.001). The vWF:Ag/ADAMTS-13 ratio increased by 57.7% before thrombosis. The difference in the vWF:Ag/ADAMTS-13 ratio from presentation to the final observation was therefore more prominent in the DVT group than in the non-DVT group (+ 3.39 vs. + 0.16, *p* = 0.026). The overall trend indicated by regression modeling was that an increased RR was associated with increased vWF:Ag and decreased ADAMTS-13.

### Plasma concentration of sP-selectin in cancer patients with and without DVT

As implied in Table 4, the concentration of sP-selectin significantly increased after chemotherapy (median 81.95 ng/mL vs. 92.5 ng/mL, *p* < 0.001), with a median of a + 7.7 ng/mL increment from baseline. In other words, though they might overlap, both platelets and endothelial cells seem to be activated by the administration of chemotherapy, as reflected by the positive delta value. However, the significance was lost when considering the magnitude of the sP-selectin increment over the chemotherapy cycle (*p* = 0.106). We also performed the same statistical analyses for sP-selectin levels determined by ELISA with another endothelial marker: the vWF:Ag level. As expected, both pre- (*r* = 0.439, *p* = 0.005, see Fig. 2c) and postchemotherapy (*r* = 0.46, *p* = 0.003, data not shown) sP-selectin levels showed a positive correlation with vWF:Ag.

### Univariate and multivariate analyses of the risk of DVT in cancer patients undergoing chemotherapy

In the univariate analysis, there were no significant associations with age at diagnosis (*p* = 0.739) or treatment with cisplatin/carboplatin agents (*p* = 0.228), fluorouracil (*p* = 0.182), anthracycline (*p* = 0.440), or steroids (*p* = 377). Five patients used an anti-vascular endothelial growth factor agent (bevacizumab), showing a negative correlation with DVT (*p* = 0.739). The incidence of DVT was not associated with sex (*p* = 0.477), cancer type (0.341), cancer stage (0.904), ABO blood group (*p* = 0.825), D-dimer level (*p* = 0.242 for cut-off 2320 U/L and *p* = 0.123 for 4622 U/L) or high Khorana risk score (*p* = 0.07). However, the following variables were significantly associated with the incidence of DVT: sP-selectin (*p* = 0.004), vWF:Ag (*p* = 0.013), and ADAMTS-13 (*p* = 0.029).

Finally, in a stepwise manner, multiple logistic regression analysis was performed using DVT incidence as the dependent variable vs. the Khorana risk score, clinical profile (D-dimer, type of chemotherapy) and three possible biomarkers (sP-selectin, vWF:Ag, and ADAMTS) starting with a full model and then removing the nonsignificant variable one by one. The potential independent variables were dichotomously categorized using a predetermined cut-off value. The final model obtained by stepwise regression analysis revealed that only vWF:Ag levels ≥2.35 IU/mL (RR 3.80; 95% CI 1.15–12.48, *p* = 0.028) and ADAMTS-13 levels ≤1.03 IU/mL (RR 2.67; 95% CI 1.12–23.82, *p* = 0.005) were related to DVT incidence, as shown in Table 5. As these variables are independent, the individual variables are multiplicative for the risk, indicating that more than one variable suggests a markedly increased risk for DVT. The Hosmer-Lemeshow statistics test for the entire model suggested a good fit, with a value of 3.349 (*p* = 0.138), and the ROC area was 0.873 (95% CI 0.675–0.925, *p* = 0.004), which indicates good differentiation [31].

**Table 5:** Univariate and multivariate analyses of the probability of predicting DVT incidence in cancer patients undergoing chemotherapy

Covariate	Univariate analysis		Multivariate analysis^**a**^	
	RR (95% CI)	***P***	RR (95% CI)	***P***
Age ≥ 55 years	1.50 (0.13–16.32)	0.739		
Male sex	2.00 (0.29–13.51)	0.477		
Type of cancer, low risk vs. high/very high risk	2.53 (0.37–17.24)	0.341		
Cancer stage, localized vs. advanced/metastasis	0.88 (0.13–6.00)	0.904		
Chemotherapy regimen				
Cisplatin/carboplatin-based	3.27 (0.47–22.46)	0.228	0.454 (0.208–26.68)	0.704
Fluorouracil-based	4.77 (0.48–46.90)	0.182	1.309 (0.606–17.14)	0.456
Bevacizumab	1.50 (0.13–16.32)	0.739		
Anthracycline	2.66 (0.22–32.17)	0.440		
Use of steroids	3.20 (0.24–42.18)	0.377		
Group O blood type	0.77 (0.77–7.71)	0.825		
D-dimer ≥2320 ng/mL (75th percentile)	2.55 (0.52–12.48)	0.242		
D-dimer ≥4622.4 ng/mL (90th percentile)	3.57 (0.707–18.04)	0.123	1.00 (0.99–2.01)	0.900
High Khorana risk score	8.07 (0.84–77.1)	0.07	0.537 (0.121–13.65)	0.537
Prechemotherapy sP-selectin ≥105.5 ng/mL	11.67 (2.22–61.27)	0.004	1.02 (0.98–11.05)	0.260
Prechemotherapy vWF:Ag ≥ 2.35 IU/mL	7.50 (1.53–36.71)	0.013	3.80 (1.15–12.48)	0.028
Prechemotherapy ADAMTS-13 ≤ 1.03 IU/mL	13.5 (1.31–38.65)	0.029	2.67 (1.22–23.82)	0.005

*NOTE*: ^**a**^In the multivariate model, the area under the ROC curve = 0.873 (95% CI 0.675–0.925, *p* = 0.004). Hosmer-Lemeshow goodness-of-fit test: X^2^ = 3.349, df = 8, *p* = 0.138. Null hypothesis = 0.5

## Discussion

DVT is a blood clot that forms within a deep vein in the body, typically in the lower extremities [1]. The occurrence of DVT, as well as thrombosis in any part of the human body, relates to Virchow’s triad, which states three primary reasons: alterations in blood flow, hypercoagulability and endothelial injury [1, 32]. This study focused on the first cause of DVT in cancer patients undergoing chemotherapy. In brief, we included forty cancer patients, of whom 5 (12.5%) developed asymptomatic DVT as early as 3 months following chemotherapy. A previous study by Blom et al. [2] also found that the risk of developing thrombosis was highest in the first 3 months after cancer diagnosis (adjusted OR 53.4, 95% CI 8.6–334.3). Similarly, another study also observed these events over a median follow-up of 2.4 months in patients treated with chemotherapy [33].

Our present study confirmed that cancer is associated with increased levels of both sP-selectin and vWF:Ag, two molecules from the endothelial WPB. Endothelial cells, megakaryocytes and platelets can synthesize vWF, the largest multimeric glycoprotein involved in regulating hemostasis; thus, a high-level of vWF:Ag is a reliable marker of thrombosis. A recent study also found high vWF expression in tumor cells [34], contributing to a significant elevation of vWF levels in cancer patients. However, the baseline ADAMTS-13 level was lower than the normal reference level, and the response after chemotherapy varied.

Multivariate logistic regression revealed two independent risk factors related to DVT in cancer patients undergoing chemotherapy: vWF:Ag and ADAMTS-13. A vWF:Ag level above the 75th percentile was associated with a 3.8-fold increased risk of developing DVT, and an ADAMTS-13 level below the 25th percentile was associated with an approximately 2.7-fold increased risk of developing DVT in cancer patients after chemotherapy. Therefore, following cancer diagnosis, patients with a vWF:Ag level greater than 2.35 IU/mL and an ADAMTS-13 level less than 1.03 IU/mL have a high probability of developing DVT during chemotherapy, suggesting that both vWF:Ag and ADAMTS-13 have a mechanistic effect. Similar studies were conducted by Pepin et al. [35] from France and Obermeier et al. [36] from Austria: these authors reported different perspectives on the role of ADAMTS-13 in relation to high vWF:Ag. Previous studies have shown that the activity and level of ADAMTS-13 are slightly lower in cancer patients than in healthy controls [37, 38]. Theoretically, a deficiency of ADAMTS-13 results in the presence of UL-vWF [39]. It is therefore believed that the circulating levels of ADAMTS-13 may influence the circulating levels of vWF and/or its function and thereby the risk of thrombosis.

We explored the effect of high sP-selectin levels and then stratified patients into 2 groups based on the 75th percentile of sP-selectin levels (cut-off point 105.5 ng/mL). Surprisingly, sP-selectin was not an independent risk factor for DVT and failed to reach statistical significance in the multivariate analysis. Several studies have demonstrated elevated levels of sP-selectin in cancer patients with DVT [40–42], inconsistent with the results of the present study. Notably, our cut-off value was higher than that in other studies by Ay et al. (53.1 ng/mL) [41] and Ramaciotti et al. (90 ng/mL) [42], partly because of the higher sP-selectin levels at baseline in our study population. This study showed that high sP-selectin levels cannot differentiate or identify cancer patients who will develop DVT early after undergoing chemotherapy.

The dynamics of plasma biomarkers should be accounted for in an absolute DVT risk assessment due to changes after chemotherapy. As shown in Fig. 3 and Table 4, temporal changes in sP-selectin, vWF:Ag, and ADAMTS-13 levels were observed following several courses of chemotherapy. There were no differences between baseline and postchemotherapy sP-selectin levels, as reflected by the nonsignificant delta value for DVT incidence (+ 6.6 vs. + 25. 4 ng/mL, *p* = 0.106, Mann-Whitney U test). However, when assessing its association with chemotherapy, there was a significant reduction in ADAMTS-13 from baseline in those who developed DVT (delta value − 0.27 with DVT vs. + 0.05, *p* = 0.015), while nearly all patients showed slightly increased vWF:Ag (delta value + 0.27 vs. + 0.21, *p* = 0.379) over time. Our findings revealed deficient levels of ADAMTS-13, which regulates the size and adhesive activity of plasma vWF. The corelationship between these two markers resulted in a wide gap in the vWF:Ag/ADAMTS-13 ratio.

Since the relation in the size of UL-vWF multimers via ADAMTS-13 is a relevant mechanism of thrombosis in cancer patients, reduced ADAMTS-13 levels as well as high plasma vWF:Ag levels, causing a high vWF:Ag/ADAMTS-13 ratio, may serve as independent predictive factors. However, there are no mechanistic or biochemical data that might explain this observed association between high vWF:Ag and low ADAMTS-13. We hypothesized several possible mechanisms for this phenomenon, including impaired protein synthesis in the liver or endothelial dysfunction associated with direct chemotherapy toxicity and consumption by increased vWF substrates [36, 43]. Various oncogenes have also been found to regulate the expression of extracellular proteinases, including matrix-degrading metalloproteinases, which can directly disrupt ADAMTS-13 activities [43]. Both the mechanisms of declination and to what extent ADAMTS-13 contributes require further study using different approaches.

In this study, we provide a piece of the puzzle regarding the cause-and-effect relationship of increased endothelial markers in cancer patients and DVT as a result of chemotherapy. In this respect, our study demonstrated a new perspective by determining that DVT in cancer patients is related to immunothrombosis. Thus, the coagulation process is not the sole mechanism that leads to thrombosis; rather, vascular inflammation on account of direct endothelial toxicity related to chemotherapy exposure may play a role. Biochemically, chemotherapy-induced VECA and vascular inflammation are indicated by increasing circulating endothelial cells and markers such as vWF:Ag and sP-selectin. The rising endothelial activities as a result of inflammation induce various changes in endothelial cells, leukocytes and platelets, promoting procoagulant and prothrombotic surfaces in blood vessel walls that increase the risk of DVT.

The paradigm above provides new insights for studies concerning novel thromboprophylaxis strategies and suggests a role for anti-inflammatory agents, which could be used for DVT prevention with a lower risk of bleeding complications than conventional therapeutic approaches.

Despite these results, there were several limitations to the study that should be addressed. First, this was a single-center study with a relatively small sample size; thus, it was not large enough for a subgroup analysis for a deeper understanding. Therefore, the current findings must be confirmed in larger, multicenter and prospective studies. Second, this study incorporated only clinical probability testing at study enrollment to exclude the lack of CUS screening for DVT. Third, we did not accounted for PE incidence in the study outcome, even though 33% of PEs are not preceded by documented DVT, especially in patient with active cancer [44]. Fortunately, no study participants showed signs or symptoms suggesting PE during the observation period. Fourth, we acknowledge that there must be residual confounding by variables not measured in our study (e.g., smoking) that can influence DVT outcome or affect the levels of the covariates. Fifth, because we assessed the antigen level rather than activity, we could not assess the functional impact of the wide gap in the vWF:Ag: ADAMTS-13 ratio. Last, we were unable to investigate the relationship between DVT and other endothelial biomarkers, such as tissue factor activity, prothrombin fragments, the level of coagulation factor VIII, the dynamics of D-dimer, hereditary thrombosis risk factors or the effect of ADAMTS-13.

## Conclusion

Our study demonstrated the following: 1) cancer is a prothrombotic stage as reflected by abnormal sP-selectin, vWF:Ag and ADAMTS-13 levels detected by ELISA; 2) following chemotherapy, patients who develop DVT show a particular pattern: high plasma levels of vWF:Ag and low plasma levels of ADAMTS-13, generating a wide gap in the vWF:Ag/ADAMTS-13 ratio; 3) by applying the 75th and 25th percentiles as cut-off points, we determined that a baseline level of vWF:Ag greater than 2.35 IU/mL and 4) an ADAMTS-13 level less than 1.03 IU/mL in cancer patients were independent risk factors for DVT after chemotherapy.

Further research is needed to understand the important role of the immune system and vascular inflammation in the pathogenesis of DVT in cancer patients undergoing chemotherapy and thus to provide insights into novel thromboprophylaxis strategies or suggest a role for anti-inflammatory agents, which could be used for DVT prevention with a lower risk of bleeding complications than conventional therapeutic approaches.

## Data Availability

Database of all patients and statistical analaysis are available upon request and authorization from Dr. Kariadi Hospital.

## Abbreviations

AML: Acute myeloid leukemia
AST: Alanine transaminase
DVT: Deep vein thrombosis
ELISA: Enzyme-linked immunosorbent assay
GFR: Glomerular filtration rate
PE: Pulmonary embolism
PSGL-1: P-selectin glycoprotein ligand 1
VTE: Venous thromboembolism
VECA: Vascular endothelial cell activation
vWF: von Willebrand factor
sP-selectin: Soluble P-selectin
ULN: Upper limit of normal
UL-vWFM: Ultra large vWF molecule
VWF:Ag: von Willebrand factor antigen
ADAMTS-13: A disintegrin and metalloproteinase with a thrombospondin type 1 motif, member 13
ROC: Receiver Operating Characteristic
RR: Relative risk
WPB: Weibel-Palade bodies
